# Single-Cell RNA-Sequencing Identifies Bone Marrow-Derived Progenitor Cells as a Main Source of Extracellular Matrix-Producing Cells Across Multiple Organ-Based Fibrotic Diseases

**DOI:** 10.7150/ijbs.98839

**Published:** 2024-09-16

**Authors:** Yu Zhong, Biao Wei, Wenbiao Wang, Junzhe Chen, Wenjing Wu, Liying Liang, Xiao-Ru Huang, Cheuk-Chun Szeto, Xueqing Yu, David J. Nikolic-Paterson, Hui-Yao Lan

**Affiliations:** 1 Departments of Medicine & Therapeutics, Li Ka Shing Institute of Health Sciences, and Lui Che Woo Institute of Innovative Medicine, The Chinese University of Hong Kong, Hong Kong, China.; 2 Departments of Nephrology and Pathology, Guangdong Academy of Medical Science, Guangdong Provincial People's Hospital, Southern Medical University, Guangzhou, China.; 3 Department of Nephrology, The Third Affiliated Hospital, Southern Medical University, Guangzhou, China.; 4 Department of Nephrology, Hubei Provincial Hospital of Traditional Chinese Medicine, Affiliated Hospital of Hubei University of Chinese Medicine, Hubei Province Academy of Traditional Chinese Medicine, Wuhan, China.; 5 Department of Clinical Pharmacy, Guangzhou Eighth People's Hospital, Guangzhou Medical University, Guangzhou, China.; 6 Department of Nephrology and Monash University Department of Medicine, Monash Medical Centre, Clayton, Victoria, Australia.

**Keywords:** Fibrosis, Extracellular matrix, ECM-producing cells, Single-cell RNA-seq, Myofibroblasts

## Abstract

Fibrosis is characterized by the aberrant deposition of extracellular matrix (ECM) due to dysregulated tissue repair responses, imposing a significant global burden on fibrosis-related diseases. Although alpha-smooth muscle actin (α-SMA/*ACTA2*)-expressing myofibroblasts are considered as key player in fibrogenesis, the origin of ECM-producing cells remains controversial. To address this issue, we integrated and analyzed large-scale single-cell transcriptomic datasets from patients with distinct fibrotic diseases involving the heart, lung, liver, or kidney. Unexpectedly, not all *ACTA2-*expressing cells were ECM-producing cells identified by expressing collagen genes; instead, the majority of ECM-producing cells were myofibroblasts and fibroblasts derived from circulating bone marrow precursor, and to a lesser extent from local pericytes and vascular smooth cells in all fibrotic diseases. This was confirmed in sex-mismatched kidney transplants by the discovery that ECM-producing cells originated from recipient, not donor, bone marrow-derived progenitor cells (BMPCs). Moreover, these BMPCs-derived ECM-producing cells exhibited a proinflammatory phenotype. Thus, bone marrow-derived proinflammatory and profibrotic fibroblasts/myofibroblasts with stem cell properties serve as a major source of ECM-producing cells and may play a driving role in tissue fibrosis across a wide range of human fibrotic diseases. Targeting these ECM-producing cells may provide a novel therapy for diseases with fibrosis.

## Introduction

Fibrosis is a consequence of dysregulated deposition of extracellular matrix (ECM) in response to tissue injury. This excessive accumulation of ECM leads to progressive disruption of tissue architecture, impairing organ function and ultimately resulting in organ failure 1, 2. Multiple organs, including the liver 3, kidney 4, 5, heart 6, and lung 7 can develop fibrosis. The global burden of fibrosis-related diseases is enormous, with an annual combined incidence rate estimated at approximately five thousand per 100,000 person-years 8. Therefore, gaining a better understanding of the cellular sources of ECM production in fibrotic tissues is crucial for identifying biomarkers and developing therapeutic interventions for fibrotic injury.

It is widely accepted that alpha-smooth muscle actin (α-SMA), encoded by *ACTA2,* expressing myofibroblast is the major cell type responsible for the production and deposition of ECM in fibrotic diseases 9, 10; however, many different cell types have been proposed as the precursors of these myofibroblasts, including fibroblasts 11, 12, pericytes 13-16, epithelial cells 17, 18, endothelial cells 19, 20, macrophages 21, adipocytes 22 and other stromal cell populations 23-25. Thus, the precise cellular origins of myofibroblasts remain controversial 26, which hampers the development of effective anti-fibrosis therapies.

The emergence of single-cell RNA sequencing (scRNA-seq) has significantly improved our understanding of cellular heterogeneity in both normal and diseased tissues 13, 27-33. In this study, we investigate whether there are common cellular sources of ECM-producing cells across a range of different human fibrotic diseases by integrating and analyzing large-scale scRNA-seq datasets from fibrotic diseases of the heart, lung, liver, or kidney.

## Results

### Single-cell atlas reveals the phenotype of extracellular matrix-producing cells across multiple organ-based human fibrotic diseases

**Figure 1:**
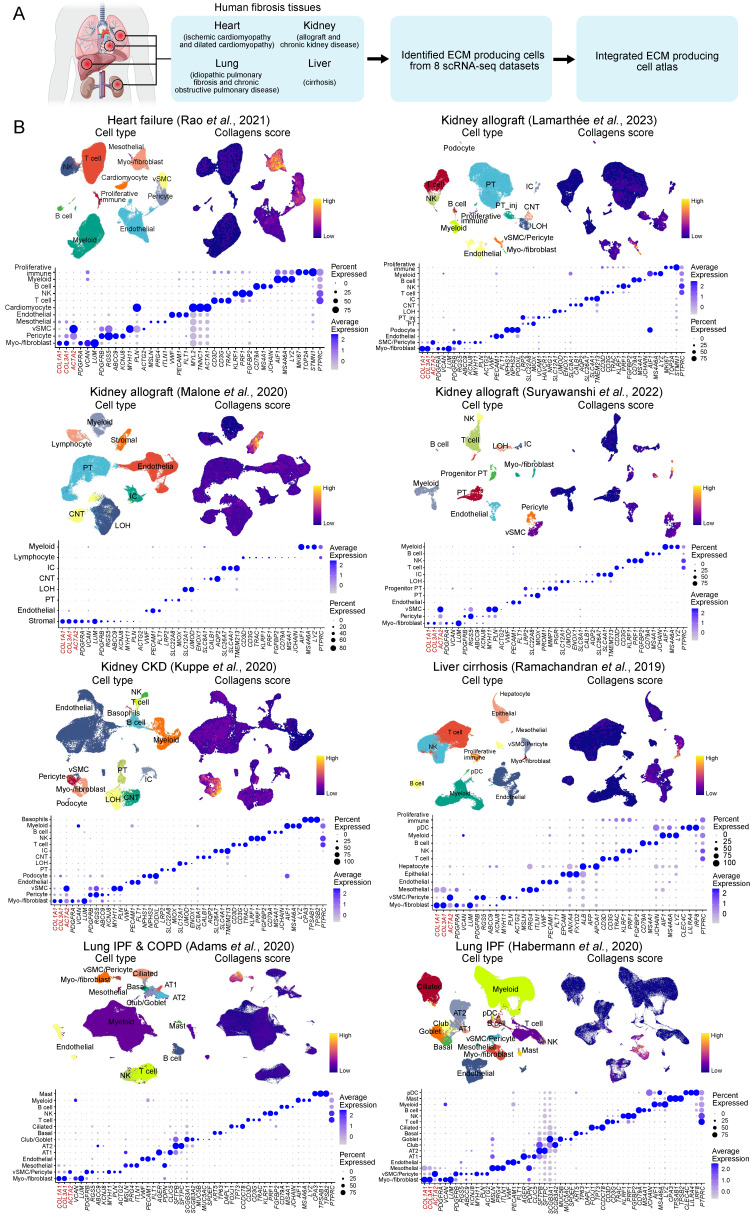
** Stromal cells exhibit high extracellular matrix production across multiple organ-based fibrotic diseases. (A)** Schematic of the study strategies for generating extracellular matrix (ECM)-producing cell atlas from human fibrosis tissues. **(B)** The upper UMAPs show the cell clusters (left panel) and the ECM score (right panel). The lower bubble plot shows the expression of canonical cell marker genes in each cell type. Bubble size indicates the proportion of cells of inferred cell type expressing each marker; color intensity represents the average expression level. vSMC, vascular smooth muscle cells; NK, natural killer cells; PT, proximal tubular cells; PT_inj, injured proximal tubular cells; IC, intercalated cells; LOH, loop of Henle; CNT, connecting tubule; pDC, plasmacytoid dendritic cells; AT1, alveolar type 1 cells; AT2, alveolar type 2 cells.

To systematically investigate the cell types responsible for ECM production in human fibrotic diseases, we analyzed eight publicly available scRNA-seq datasets of human fibrotic diseases involving the heart, kidney, liver, and lung 13, 27-33 **(Figure 1A, Table S1-7)**. For each dataset, we conducted quality control and cell clustering to identify distinct cell populations. Cell types in each dataset were annotated according to representative markers** (Figure 1B, Table S8)**. To elucidate the specific cell types contributing to the production of ECM in fibrotic tissues, we quantified the ECM score by assessing the expression levels of collagens, glycoproteins, and proteoglycans 34. Notably, stromal cells including myofibroblasts/fibroblasts (myo-/fibroblast), vascular smooth muscle cells (vSMC), pericytes, and mesothelial cells consistently exhibited higher ECM scores across all datasets **(Figure 1B)**. Furthermore, these stromal cells demonstrated elevated expression levels of fibrosis-related collagen genes *COL1A1* and *COL3A1* compared to other cell types **(Figure 1B),** suggesting that these stromal cells serve as the primary source for ECM production in fibrotic tissues.

**Figure 2:**
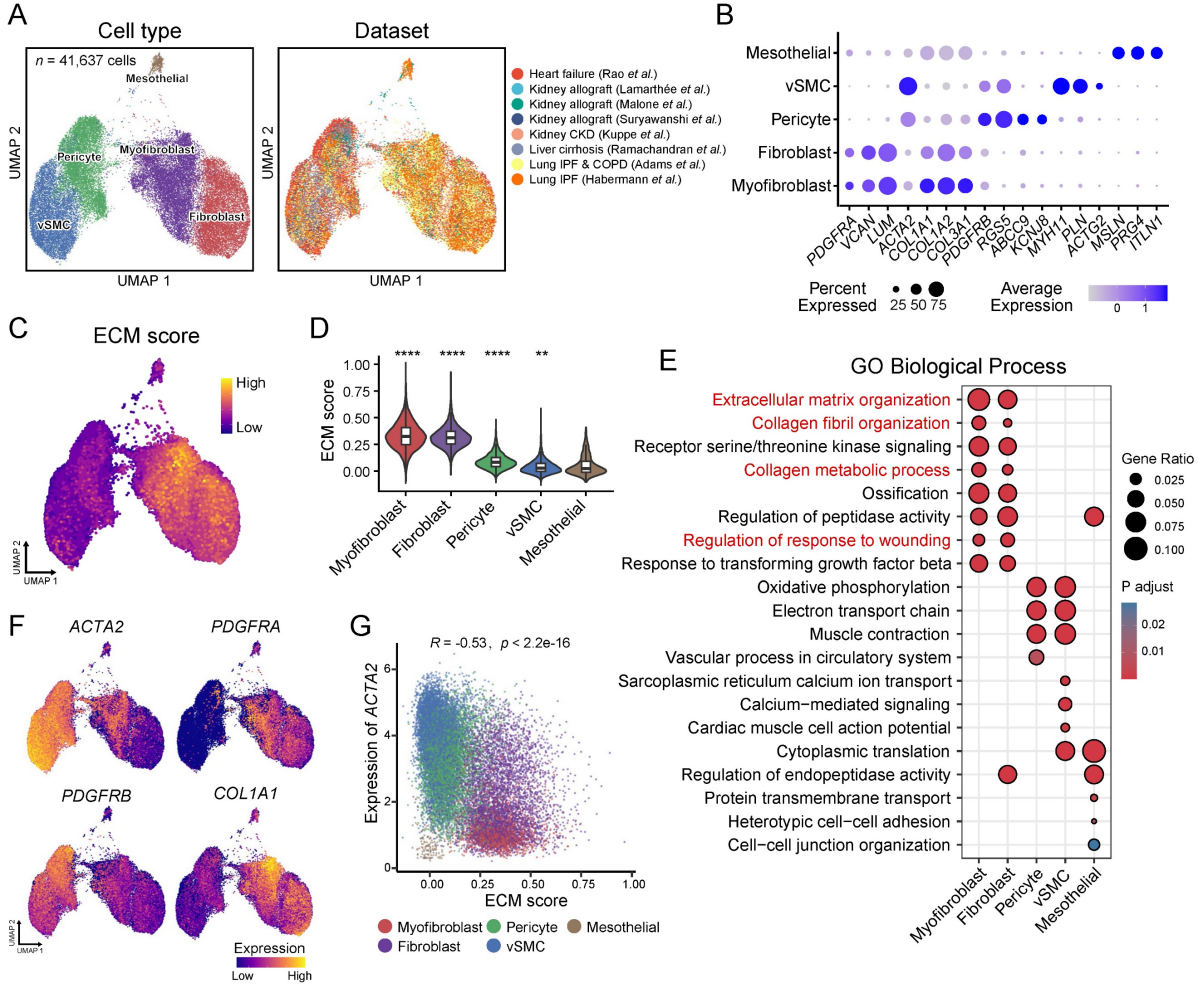
** Atlas of ECM-producing cells in fibrosis tissues. (A)** UMAP of clusters (left panel) of ECM-producing cells and the source of datasets (right panel). vSMC, vascular smooth muscle cells**. (B)** Bubble plot shows canonical cell marker gene expression in each cell type. Bubble size indicates the proportion of cells expressing each marker; color intensity represents the average expression level. **(C)** UMAPs of cells colored by gene score of ECM genes. **(D)** Violin plot displays the ECM scores in different ECM-producing cell types. Statistical analysis was performed with mesothelial cells as control using Wilcoxon test.** (E)** Comparing Gene Ontology (GO) enrichment among different cell types. **(F)** UMAPs of cells colored by gene expression of feature genes. **(G)** Scatter plot of expression of *ACTA2* and ECM score among different cell types. Statistical analysis was performed using Student's t-test. * *p* < 0.05, ** *p* < 0.01, *** *p* < 0.001 and ****p < 0.0001.

To focus on ECM-producing cells, we integrated these stromal cells, including myo-/fibroblast, vSMC, pericytes, and mesothelial cells (41,637 cells), with adjustment for cross-dataset batch effects, and finally generated an atlas of ECM-producing cells in human fibrotic diseases. We identified five main cell types responsible for ECM production **(Figure 2A and Figure S1A, Table S9)**, including fibroblasts and myofibroblasts that exhibited high expression of *PDGFRA*, *VCAN,* and* LUM,* as well as collagen genes *COL1A1*, *COL2A1* and* COL3A1* **(Figure 2B)***. ACTA2* (encoding alpha-smooth muscle actin) was highly expressed by vSMC and pericytes, while *ACTA2* was expressed at lower levels by myofibroblasts and fibroblasts **(Figure 2B)**. However, *ACTA2* displayed higher expression in myofibroblasts compared to fibroblasts. Pericytes were identified by expression of *PDGFRB*, *RGS5*, *ABCC9* and *KCNJ8*; vSMC were identified by expression of *MYH11*, *PLN* and *ACTG2;* and mesothelial cells were identified by expression of *MSLN*, *PRG4* and *ITLN1* **(Figure 2B)**. Although these stromal cells showed a higher ECM score than other cell types, both myofibroblasts and fibroblasts exhibited significantly higher ECM score than pericytes, vSMC and mesothelial cells **(Figure 2C-D and Figure S1B-D)**. Furthermore, functional enrichment analysis revealed insights into the distinct roles played by different stromal cell types **(Figure 2E)**. Myofibroblasts and fibroblasts were primarily involved in ECM organization, collagen fibril organization, collagen metabolic process and regulation of response to wounding **(Figure 2E)**. On the other hand, pericytes and vSMC participated in oxidative phosphorylation and muscle contraction while mesothelial cells were more related to cell adhesion functions **(Figure 2E)**.

Although *ACTA2* has been considered as a hallmark of the transition of quiescent fibroblasts to an activated phenotype with synthesis of ECM components 35, the highest expression levels of *ACTA2* were observed in pericytes and vSMCs which were marked by high expression of *PDGFRB* but low expression of *COL1A1* **(Figure 2F)**. However, myofibroblasts and fibroblasts, which express high levels of collagen genes and *PDGFRA*, exhibited lower expression of *ACTA2* compared to pericytes and vSMCs** (Figure 2F)**. Interestingly, there was a significant negative correlation between the expression of *ACTA2* and ECM score **(Figure 2G)**. These findings indicate that *ACTA2* is not a precise marker for identifying ECM-producing cells in fibrotic tissues. Previous studies have found that *ACTA2* is an inconsistent marker of cells producing type I collagen and other ECM proteins 36, 37.

Overall, a comprehensive single-cell atlas containing multiple human fibrotic diseases demonstrates that not all *ACTA2*-expressing cells are ECM-producing cells; instead myofibroblasts and fibroblasts are the primary cell types responsible for ECM deposition in fibrotic tissues.

### Heterogeneity within ECM-producing myofibroblasts and fibroblasts

**Figure 3:**
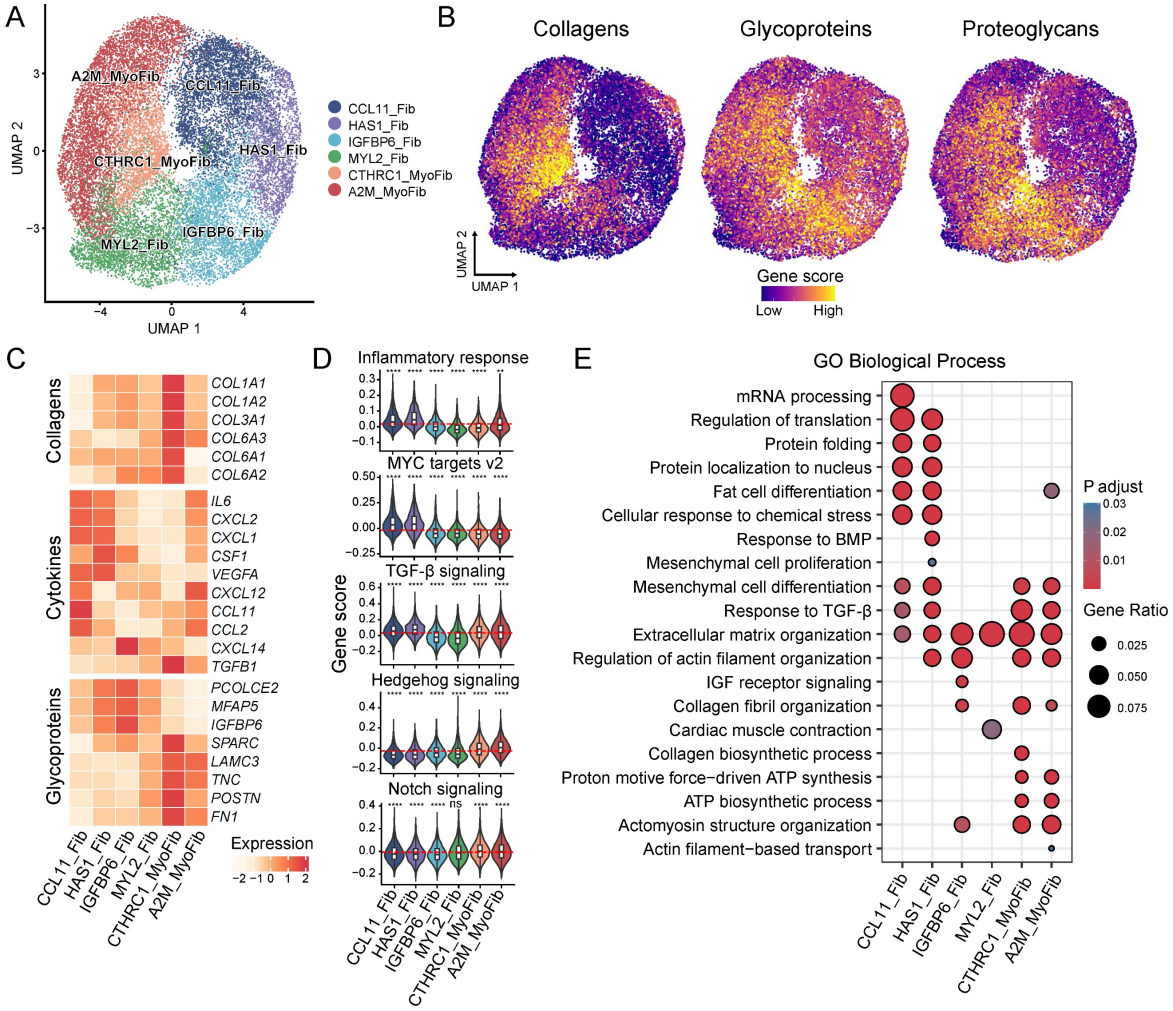
** Heterogeneity of myo-/fibroblasts. (A)** UMAP shows the six identified cell subclusters of myo-/fibroblasts. **(B)** UMAPs show the gene scores for collagens, glycoproteins, and proteoglycans. **(C)** The heatmap displays the expression of selected genes for collagens, cytokines, and glycoproteins. **(D)** Violin plot displays the gene scores of myo-/fibroblasts cell subtypes. Each subtype is compared to the mean of all cells and the red dashed line indicates the mean of the gene score. Statistical analysis was performed using the Wilcoxon test.** (E)** GO enrichment among the different myo-/fibroblasts cell types. * *p* < 0.05, ** *p* < 0.01, *** *p* < 0.001, **** *p* < 0.0001**.**

Reclustering of ECM-producing myofibroblasts and fibroblasts identified six subtypes which were annotated according to the dominant cluster-specific genes: *CCL11*+ fibroblast (CCL11_Fib), *HAS1*+ fibroblast (HAS1_Fib), *IGFBP6*+ fibroblast (IGFBP6_Fib), *MYL2*+ fibroblast (MYL2_Fib), *CTHRC1*+ myofibroblast (CTHRC1_MyoFib) and *A2M*+ myofibroblast (A2M_MyoFib) **(Figure 3A and Figure S2A-C, Table S10)**. The expression of ECM components also showed heterogeneity among these subtypes. The two myofibroblast subtypes, CTHRC1_MyoFib and A2M_MyoFib, demonstrated higher expression of collagen genes such as *COL1A1, COL1A2* and *COL3A1*, which are crucial for wound healing and tissue regeneration **(Figure 3B-C and Figure S2C)**. Glycoprotein and proteoglycan gene expression were more highly enriched in IGFBP6_Fib and CTHRC1_MyoFib **(Figure 3B)**. Besides, MYL2_Fibs showed high expression levels of proteoglycan genes as well **(Figure 3B)**.

In addition to the deposition of ECM, the release of cytokines also plays a crucial role in the inflammation-fibrosis process 1. The proinflammation gene *IL6* and vascular endothelial growth factor-A (*VEGFA*) were found to be highly expressed in the CCL11_Fib and HAS1_Fib **(Figure 3C)**. Homeostatic chemokines responsible for myeloid cell recruitment, such as *CXCL1*, *CXCL2*, *CXCL12*, *CCL11* and *CCL2* exhibited elevated expression levels in CCL11_Fib 38 **(Figure 3C)**. Moreover, CCL11_Fib also expressed *CSF1* which is responsible for macrophage recruitment, proliferation, and polarization 39 **(Figure 3C)**. These results indicate that the CCL11_Fib subtype has a pronounced proinflammatory phenotype in fibrotic tissues. *TGFB1,* a well-studied fibrogenic cytokine 8, displayed high expression in CTHRC1_MyoFib consistent with the observed increased production of ECM by this subtype **(Figure 3B-C and Figure S2D)**.

The gene score of the inflammatory response showed the highest levels in CCL11_Fib and HAS1_Fib **(Figure 3D)**, providing further evidence of the proinflammatory function of these fibroblast subtypes. MYC plays an important role in cell proliferation, as well as the maintenance of stemness and pluripotency 40. The target genes regulated by MYC also showed a high gene score in CCL11_Fib and HAS1_Fib **(Figure 3D)**.

In addition, CCL11_Fib and HAS1_Fib were enriched for biological processes related to the regulation of translation, protein folding, and mesenchymal cell differentiation **(Figure 3E)**, indicating their high levels of cell proliferation and stemness. TGFB1 signaling was enriched in CCL11_Fib, HAS1_Fib, CTHRC1_MyoFib and A2M_MyoFib subtypes **(Figure 3D-E)**. Hedgehog and Notch signaling, known for their crucial roles in the activation of fibroblasts during fibrosis 41, displayed high gene scores in CTHRC1_MyoFib and A2M_MyoFib **(Figure 3D)**. Meanwhile, collagen fibril organization as well as ATP biosynthetic process were enriched in CTHRC1_MyoFib and A2M_MyoFib **(Figure 3E)**, suggesting their involvement in ECM deposition and cell contractility functions. Our findings highlight the heterogeneity within ECM-producing myofibroblasts and fibroblasts by identifying distinct molecular signatures and functional characteristics for each subtype. This knowledge contributes to a better understanding of their respective roles in tissue remodeling during fibrosis development.

### The origins of ECM-producing cells in fibrotic tissues

**Figure 4:**
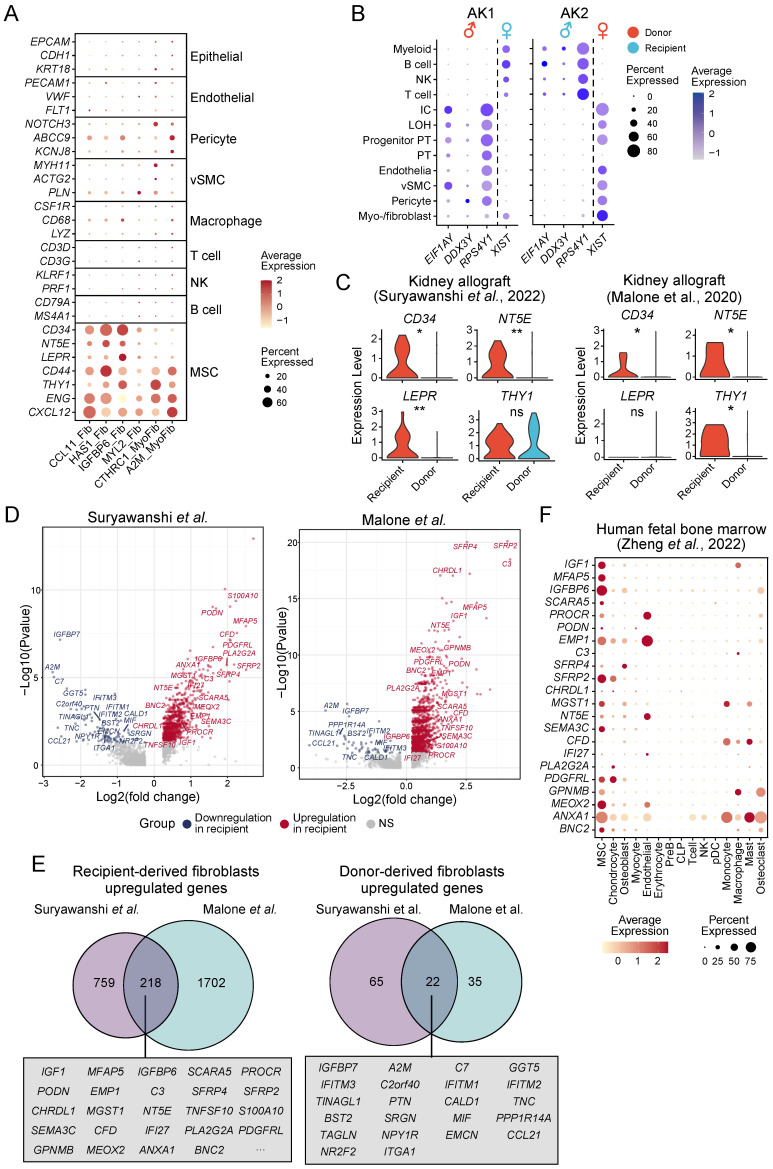
** Infer the potential cellular origins of myo-/fibroblasts subtypes. (A)** Bubble plot shows expression of cell type specific markers in myo-/fibroblasts subtypes. **(B)** Bubble plot shows the annotation of recipient/donor origin of cells based on female X chromosome (*XIST*) and male Y chromosome (*RPS4Y1*, *EIF1AY*, and *DDX3Y*) gene expression. **(C)** Violin plot displays the expression of HSC and MSC markers in recipient-derived and donor-derived fibroblasts. Statistical analysis was performed using Wilcoxon test. **(D)** Volcano plots illustrate differentially expressed genes of recipient-derived fibroblasts compared with those of donor-derived fibroblasts.** (E)** Venn diagram shows the overlap of marker genes of recipient-originating and donor-originating fibroblasts between two kidney allografts datasets. Top rank fold change overlapped genes show in box. **(F)** Bubble plot shows expression of recipient-originating fibroblast markers in each cell type from human fetal bone marrow. MSC, mesenchymal stem cells; PreB, B cell progenitors; CLP, common lymphoid progenitors; pDC, plasmacytoid dendritic cells; NK, natural killer cells. * *p* < 0.05, ** *p* < 0.01, *** *p* < 0.001 and ****p < 0.0001.

To identify the cellular origins of the different ECM-producing myofibroblasts and fibroblasts subtypes, we analyzed the expression of lineage-specific markers in these populations. The results revealed minimal expression of epithelial, endothelial or lymphocyte (T cells, natural killer cells, and B cells) lineage markers in ECM-producing myofibroblasts and fibroblasts across all four major fibrotic organs **(Figure 4A)**, indicating that these specific cell types do not significantly contribute to the pool of ECM-producing cells in fibrotic tissues. However, a majority of ECM-producing cells exhibited high expression of hematopoietic stem cell (HSC) and mesenchymal stem cell (MSC) markers such as *CD34*, *NT5E* (CD73), *LEPR*, *CD44*, *THY1* (CD90), *ENG* (CD105), and *CXCL12* 42; while only low levels of expression of pericyte, vSMC, and macrophage lineage markers were observed in these cells **(Figure 4A)***.* The consistent expression of HSC and MSC markers in ECM-producing cells across various datasets suggest a conserved fibrotic mechanism, despite differences in tissue origin or potential stages of fibrosis **(Figure S3A)**. However, in the kidney datasets from Malone *et al*. and Kuppe *et al*., only a small subset cells (HAS1_Fib and IGFBP6_Fib) exhibited expression of HSC and MSC markers (*CD34*, *MT5E/CD73*, and *LEPR*). This observation is consistent with low proportions observed for myo-/fibroblast subsets CCL11_Fib, HAS1_Fib, and IGFBP6_Fib in these two datasets **(Figure S2B and S3A)**. These results indicate that HSCs or MSCs serve as the primary cellular source for ECM-producing cells and provide partial clarification regarding the controversial origins of ECM-producing cells in fibrotic tissues.

To determine whether ECM-producing cells are derived from either tissue-resident cells or circulating cells, we identified myo-/fibroblasts in a previous study of human kidney allograft biopsies 29 **(Figure S3B, Table S5)**. In this study, Suryawanshi *et al.* took advantage of the recipient-donor sex mismatch revealed by the expression of X and Y chromosome-specific genes to distinguish fibroblasts originating from either the recipient or donor. The use of inherent lifelong sex gene markers (X and Y chromosomes) for identifying the origin of ECM-producing cells is an innovative approach, which provides a definitive and unalterable means of tracing cell lineage. Myofibroblasts and fibroblasts were categorized as either recipient- or donor-derived based on the expression of sex-specific genes **(Figure 4B)**. It was observed that in a transplant with tubulointerstitial fibrosis and worsening graft function (AK1), more than half of the ECM-producing myo-/fibroblasts (78.8%) were recipient-derived, whereas only a small proportion of ECM-producing cells (8.1%) were derived from recipient in the second kidney transplant without fibrosis (AK2), demonstrating that bone marrow-derived myo-/fibroblasts contribute to the development of renal fibrosis in patients with chronic renal allograft rejection. Other ECM-producing cells, such as pericytes and vSMC, were entirely donor-derived **(Figure 4B)**. We noted that recipient-derived myo-/fibroblasts showed significantly higher expression of HSC and MSC markers, which was further validated in another kidney allograft dataset published by Malone *et al*. 28 **(Figure 4C)**. These findings suggest that these recipient-derived fibroblasts may originate from circulating bone marrow-derived progenitor cells (BMPCs) and are responsible for ECM production.

The marker genes of recipient-derived and donor organ-derived fibroblasts were identified through differential gene expression analysis, revealing a significant overlap between the two kidney allograft datasets **(Figure 4D-E, Table S11-12)**. In a scRNA-seq dataset of human fetal bone marrow 43, we validated that the top differentially expressed overlapping markers of recipient-derived fibroblasts showed high expression in bone marrow-derived MSC **(Figure 4F and Figure S3C-D)**.

**Figure 5:**
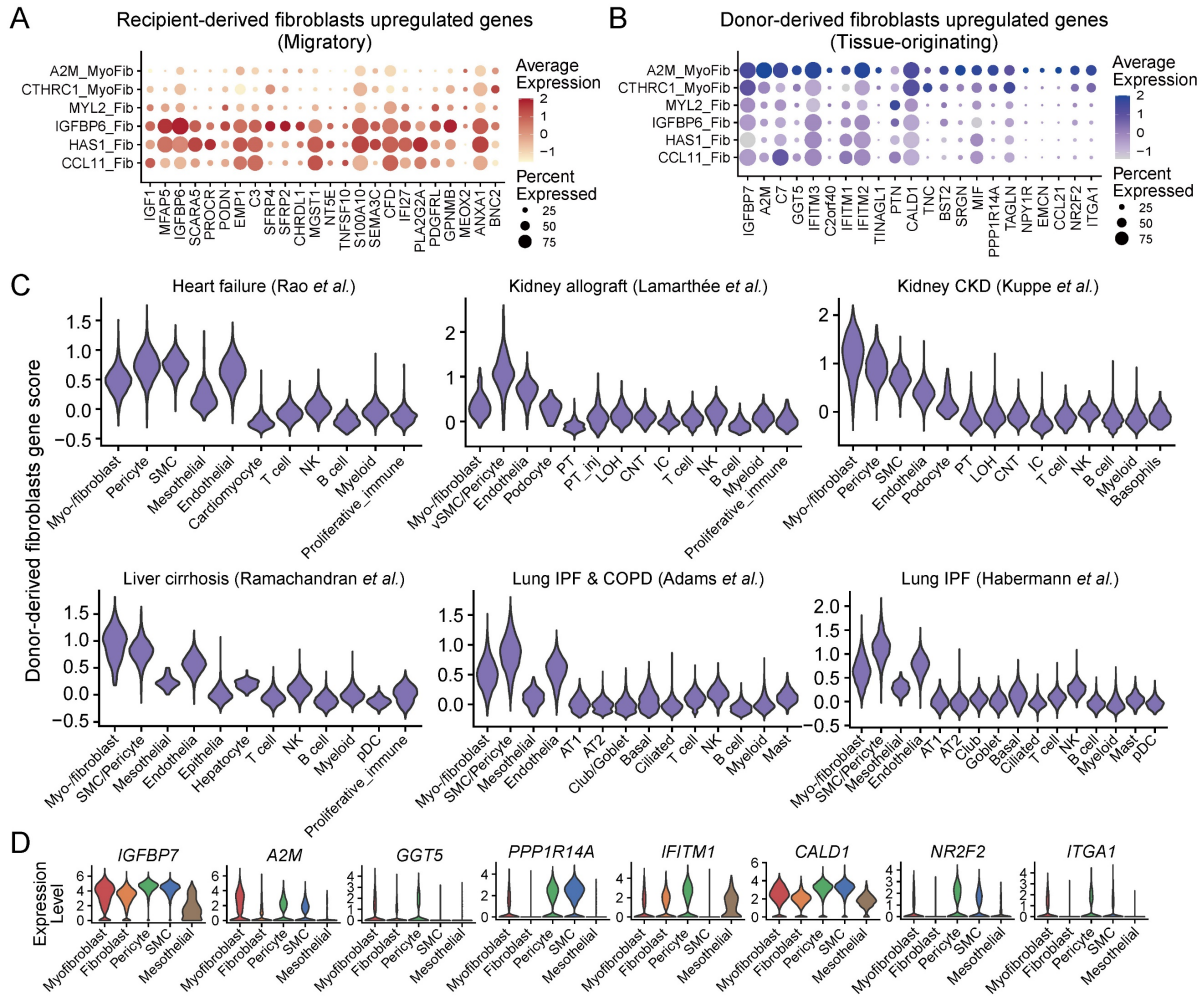
** Analysis of the cellular source of ECM-producing cells in fibrotic tissues. (A-B)** Bubble plots show expression of recipient-originating fibroblast markers **(A)** and donor-originating fibroblast markers** (B)** in myo-/fibroblasts subtypes.** (C)** Violin plot displays the gene score of donor-originating fibroblast markers in different cell types across six different studies. **(D)** Violin plot displays expression of donor-derived fibroblast markers in different cell types.

Furthermore, the three fibroblast subtypes; CCL11_Fib, HAS1_Fib, and IGFBP6_Fib also exhibited higher expression of recipient-derive fibroblast markers, which was consistent with the elevated expression of HSC and MSC markers in these cell subtypes **(Figure 5A and 4A)**. This suggests a direct connection between recipient-derived fibroblasts and progenitor cells derived from bone marrow. However, the myofibroblast subtype A2M_MyoFib showed higher expression of donor-derived (originating within the donor kidney allograft) fibroblast markers **(Figure 5B)**. Thus, it appears that this particular myo-/fibroblast subtype may have originated from tissue-resident cells rather than from circulating cells. In addition, pericytes showed the highest gene score for tissue-originating fibroblasts across multiple datasets except for myo-/fibroblasts **(Figure 5C-D)**. Recent studies have demonstrated that pericytes are a major source of myofibroblasts in chronic kidney disease 13. We discriminated the source of ECM-producing myo-/fibroblasts based on the gene score of circulating (recipient-derived) and resident (donor-derived) fibroblasts and demonstrated that 61.91% ECM-producing myo-/fibroblasts originate from the peripheral circulation, while 14.79% are derived from local tissue **(Figure S3E)**. Consistently, statistical analysis revealed that CCL1_Fib, HAS1_Fib, and IGFBP6_Fib fibroblast subtypes predominantly originated from the circulating cells, whereas A2M_MyoFib is primarily sourced from local tissue **(Figure S3F)**. These results imply that the majority of ECM-producing cells with myo-/fibroblasts phenotype originate from peripheral circulating cells, whereas a minority of fibrogenic cells derive from tissue-resident pericytes.

Collectively, we have examined the distinct cellular characteristics of ECM-producing cells originating from the local tissue and from circulating bone marrow (migratory) cells and identified that the majority of ECM-producing cells are derived from circulating BMPCs with proinflammatory phenotype. Moreover, our results highlight the innovative use of sex gene markers to determine the origin of ECM-producing cells, which surpasses current immunologic and transgenic strategies, offering higher precision and reliability.

### Cell differentiation trajectory of ECM-producing cells

**Figure 6:**
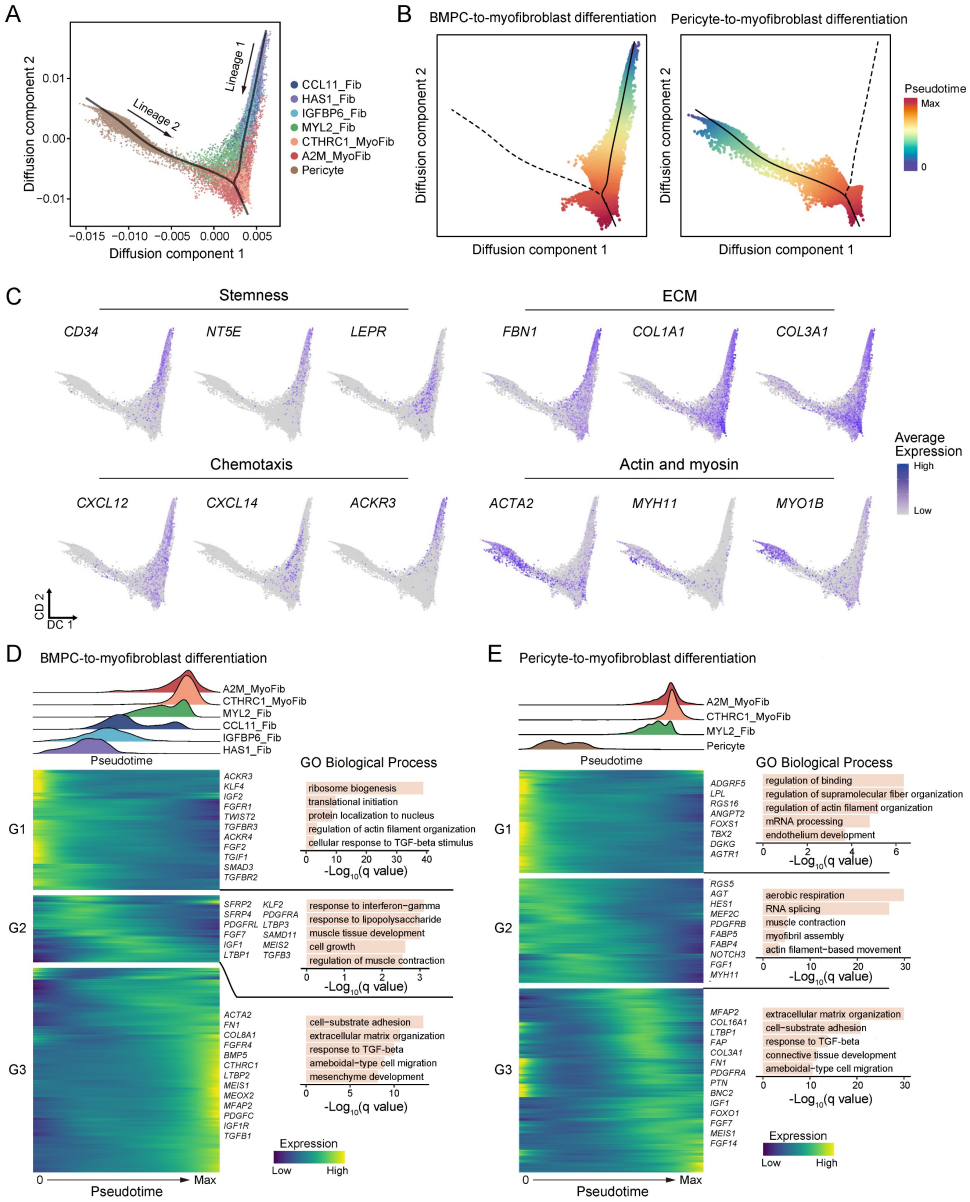
** Dynamics of ECM-producing cells differentiation. (A)** Diffusion map shows the trajectory of fibroblasts, myofibroblasts and pericytes. The black lines represent two lineage trajectories predicted by Slingshot. **(B)** Diffusion map shows the inferred pseudotime of two lineage trajectories. **(C)** The expression of selected genes on the two lineage trajectories. **(D-E)** The distribution of cell types within the pseudotime analysis of the two lineage trajectories is inferred in **(B)** (upper panel). The heatmaps display the dynamic expression changes of genes along the pseudotime and the bar plot shows the functional enrichment of gene clusters (lower panel).

We next used diffusion pseudotime analysis to examine the differentiation trajectories and dynamic molecular changes occurring within ECM-producing myofibroblasts, fibroblasts and pericytes (potential precursor for A2M_MyoFib) **(Figure 6A)**. Two distinct cell differentiation trajectories were identified: (lineage 1) BMPC-derived fibroblast-to-myofibroblast differentiation, and (lineage 2) pericyte-to-myofibroblast differentiation, which corresponded to circulating bone marrow-derived and tissue-originating sources of ECM-producing myofibroblasts, respectively **(Figure 6A-B)**.

During the differentiation of BMPC-derived fibroblasts to myofibroblasts, there was a decrease in the expression of stem cell markers, while ECM genes and *ACTA2* were elevated, indicating a differentiation trajectory from fibroblasts with high stemness and low-ECM to myofibroblasts with high-ECM **(Figure 6C)**. Moreover, chemotaxis genes *CXCL12*, *CXCL14*, and *ACKR3* were predominantly expressed in the circulating bone marrow lineage but decreased with differentiation to myofibroblasts **(Figure 6C).** This result suggests that the circulating bone-marrow lineage contributes to inflammation by recruiting immune cells to the site of tissue injury, and the migration of circulating progenitor of fibroblasts into fibrotic tissue may occur via the *CXCL12-ACKR3* (CXCR7) axis. On the other hand, the pericyte-to-myofibroblast lineage showed high expression of actin and myosin genes, but lower expression of ECM genes compared to the circulating bone marrow lineage **(Figure 6C)**. In short, circulating bone marrow-derived myo-/fibroblasts play a key role in inflammation and ECM deposition in fibrotic tissues, while tissue-originating myofibroblasts contribute more to contractile function rather than ECM deposition.

During the BMPC to myofibroblast transition, HAS1_Fib and IGFBP6_Fib subtypes were placed at the initial stage, followed by CCL11_Fib and MYL2_Fib subtypes, and completing the transition with myofibroblast subtypes CTHRC1_MyoFib and A2M_MyoFib **(Figure 6D, Table S13)**. The heatmap of differentially expressed genes demonstrates a programmed change in gene expression during BMPC to myofibroblast differentiation **(Figure 6D)**. These genes were categorized into three temporal patterns. Genes related to ribosome biogenesis, protein localization to nucleus, cellular response to TGF-β stimulus and regulation of actin filament organization were predominantly enriched at the early stage of BMPC-myofibroblast differentiation (G1) **(Figure 6D)**. Transcription factors (TFs) *KLF4*, *TWIST2*, and *SMAD3*; growth factors *FGF2* and *IGF2*; as well as receptor tyrosine kinases *FGFR1*, *TGFBR2* and *TGFBR3* showed initial expression at the early differentiation stage **(Figure 6D)**. KLF4 has been reported to regulate TGFB1-SMAD signaling pathway leading to ECM synthesis in PDGFRB^+^ cells in the lung 44. SMAD3 is a downstream effector of the TGFB1 signaling pathway, which is crucial for the fibrotic response 45. These findings suggest that these TFs and growth factors play a crucial role in initiating the differentiation process. Inflammatory processes such as response to interferon-gamma and response to lipopolysaccharide; as well as muscle tissue development and cell growth were enriched during BMPC to myofibroblast differentiation (G2) **(Figure 6D)**. TFs *KLF2* and *MEIS2*; receptor tyrosine kinases *PDGFRA* and *PDGFRL*; growth factors *FGF7*, *IGF1* and *TGFB3*; as well as latent-transforming growth factor beta-binding protein *LTBP1* and *LTBP3* were upregulated in the middle phase of the differentiation trajectory. At the later stage of differentiation, processes including cell-substrate adhesion, ECM organization, response to TGF-β and mesenchyme development became enriched (G3)** (Figure 6D)**. Meanwhile, the expression levels of specific genes and factors that play crucial roles in fibrosis such as *ACTA2,* ECM genes *FN1, COL8A1*, *LTBP2* and *MFAP2,* as well as profibrotic factors *PDGFC* and *TGFB1* were elevated in the late stage of BMPC to myofibroblast differentiation.

During pericyte to myofibroblast differentiation, pericytes served as the root of the trajectory, followed by MYL2_Fib and then the two subtypes of myofibroblasts **(Figure 6E, Table S14)**. Notably, only the fibroblast subtype MYL2_Fib participates in pericyte-to-myofibroblast differentiation, which is consistent with the low expression of recipient-originating fibroblast markers in this subtype **(Figure 6E and Figure 5A)**. At the early stages of pericyte to myofibroblast differentiation, genes associated with the regulation of binding, regulation of actin filament organization and endothelium development were enriched **(Figure 6E)**.

These biological processes reflect the characteristics of pericytes that closely interact with interstitial capillary endothelial cells and regulate capillary permeability through cell contraction. TFs *FOXS1* and *TBX2* as well as pro-angiogenic genes (*ANGPT2*, *AGTR1* and *DGKG*) 46 showed high expression at the early stage of pericyte-to-myofibroblast differentiation. A previous study has reported that TBX2^+^ cells represent a multi-potent mesenchymal population 47. As pseudotime progressed, biological processes related to actin filament-based movement, myofibril assembly and muscle contraction biological processes were enriched during the middle stage **(Figure 6E)**. Transcription factor *HES1*, growth factor *FGF1* and fatty acid-binding proteins (*FABP4* and *FABP5*) were upregulated at the middle phase of the differentiation trajectory. Towards the end of the differentiation trajectory, there was an enrichment of biological processes related to extracellular matrix organization and response to TGF-beta pathways **(Figure 6E)**. Meanwhile, several ECM genes (*MFAP2*, *COL16A1*, *LTBP1*, *FAP*, *COL3A1* and *FN1*) exhibited elevated expression levels **(Figure 6E)**. These findings suggest a stepwise progression from early regulatory events involving in pericyte to myofibroblast differentiation and finally culminating in extracellular matrix remodeling mediated by TGF-beta signaling pathway activation. Overall, we identified two distinct differentiation trajectories for ECM-producing myofibroblasts and described the transcriptional dynamics and key regulators of these processes.

## Discussion

In this study, we integrated large-scale scRNA-seq datasets derived from human fibrotic disease tissues from patients with chronic heart failure, kidney allograft and CKD kidneys, liver cirrhosis, and lung COPD and IPF. Pathologically, fibrosis is characterized by excessive ECM deposition within the tissues/organs, accompanied by the loss of normal tissue/organ structures and functions. Thus, fibrosis is a key pathological feature of chronic organ failure commonly found in the heart, lung, liver, and kidney. Although the causes leading to the development of these end-organ diseases may vary among them, excessive ECM accumulation is a unique feature. Thus, identifying the origin of ECM-producing cells is the first step towards the development of effective therapy for chronic organ diseases.

Myo-/fibroblasts were identified as the main cellular source of ECM. These ECM-producing cells were marked by *PDGFRA*, *VCAN* and *LUM.* Upon activation by its ligands like platelet-derived growth factor (PDGF), PDGFRα signaling pathway promotes the proliferation, migration, and activation of fibroblast, leading to their differentiation into myofibroblasts 48. Targeting PDGFRα signaling pathway has emerged as a potential therapeutic strategy for the treatment of fibrotic diseases. Recent preclinical and clinical studies have investigated inhibitors like tyrosine kinase inhibitor Nintedanib that targets PDGFRα signaling to attenuate fibroblast activation, ECM production, and fibrosis progression 49, 50. Despite *ACAT2* being widely used as a key marker for ECM-producing cells in tissue fibrosis, *ACTA2* expression levels exhibited a negative correlation with ECM score across ECM-producing cells, exemplified by high *ACTA2* levels in low ECM-producing pericytes and vSMC. These findings support previous studies which have found *ACTA2* to be a poor marker of collagen-producing cells in tissue fibrosis 36, 37. Therefore, the use of α-SMA/*ACTA2* has limitations as a biomarker for studying tissue fibrosis across human fibrotic diseases.

The distinct molecular features were identified in myo-/fibroblast subtypes. Myofibroblast subtype CTHRC1_MyoFib showed the highest expression of collagens genes, which is consistent with previous studies that annotated fibroblasts with high expression of *COL1A1* and *CTHRC1* as 'pathological fibroblasts' contributing to the leading edge of fibrosis in the lung 37, 51. Moreover, an inflammatory fibroblast subtype, CCL11_Fib, was identified with high expression of chemokines. In the present study, we focus on conserved fibroblast subtypes across different tissues to perform tissue repair and promote fibrosis. Although the ECM scores were various in different tissues and diseases, future studies for identifying tissue- or disease-specific markers of myo-/fibroblasts are crucial for understanding the mechanisms underlying fibrosis and for developing targeted therapeutic strategies.

Our study provides valuable insights into the origins of ECM-producing cells in fibrotic tissues. Increasing evidence shows that bone marrow-derived myo-/fibroblasts contribute significantly to fibrosis 52-56. In addition, one study has shown that a population of fibroblasts and their precursors are derived from hematopoietic stem cells 57. We identified the markers of circulating bone marrow-derived and local tissue-derived fibroblasts in kidney allografts, which provide a set of markers to discriminate the source of fibroblasts. We demonstrated that ECM-producing cells in fibrotic tissues were mainly derived from bone marrow precursors with highly expressing stem cell markers, and only partially from pericytes. The absence of fibrocyte and macrophage lineage gene markers in our ECM-producing myo-/fibroblasts subpopulations suggests that the expression of CD45 or CD68 gene markers may be lost during cell differentiation to ECM-producing cells, which may need further investigation.

Unlike immunologic and transgenic markers, sex chromosomes are immutable and consistently present in all cells, providing a lifelong marker with higher specificity and reduced ambiguity for cell lineage tracing. By using X and Y gene markers in the recipient-donor sex mismatch kidney transplants, we identified the markers of circulating bone marrow-derived (recipient-originating) and local tissue-derived (donor-originating) fibroblasts in kidney allograft scRNA-seq datasets, which provide a set of markers to discriminate the source of fibroblasts. Thus, the use of sex gene markers is highly innovative, reliable, and specific for studying the origin of cells under different physiological and pathological processes.

In our study, donor-originating fibroblast markers showed a high expression in A2M_MyoFib and pericytes. Additionally, the dataset from the study of Kuppe *et al.* 13 showed a high proportion of A2M_MyoFib subtypes (**Figure S2B**), which is consistent with their finding that tissue-resident pericytes were the main origin of myofibroblasts in chronic kidney disease. We constructed cell differentiation trajectories of ECM-producing cells and revealed two lineages for myofibroblasts which were derived from BMPC to myofibroblast differentiation and pericyte to myofibroblast differentiation. Our study provides evidence supporting both peripheral circulating BMPC and tissue-resident pericytes as important contributors to the pool of myofibroblasts within fibrotic tissues. BMPC-derived myo-/fibroblast showed much higher ECM production and a proinflammatory phenotype compared to the pericyte-derived lineage. CXCL12, also known as stromal cell-derived factor-1 (SDF-1), binds to the ACKR3 (CXCR7). Interestingly, we found that chemotactic factors *CXCL12* and *ACKR3* (CXCR7) were highly expressed in BMPC-derived myo-/fibroblast. Recent studies on laryngotracheal stenosis have demonstrated that CXCR7 is increased in laryngotracheal stenosis and promotes the proliferation and migration of fibroblasts 58, 59. This suggests that *CXCL12*-*ACKR3* plays a significant role in recruiting peripheral circulating progenitors of myo-/fibroblasts into injury sites with higher concentrations of CXCL12 (SDF-1). Therapeutic strategies that inhibit the binding of CXCL12 to ACKR3 can prevent the recruitment of MSCs to fibrotic tissues. This can be achieved using small molecule inhibitors, neutralizing antibodies, or antagonist peptides that specifically target CXCL12 or ACKR3. By obstructing bone marrow-derived myo-/fibroblast recruitment, these therapies have the potential to reduce the presence of proinflammatory and ECM-producing myo-/fibroblasts in the fibrotic tissue, thereby decreasing ECM deposition and fibrosis progression.

The dynamics of transcription factors, growth factors and functional features were described along the differentiation trajectories **(Figure 6D-E)**. During the process of BMPC-derived fibroblast to myofibroblast differentiation, several genes related to TGF-β (*TGFB1, TGFBR2, TGFBR3, LTBP1, LTBP2, LTBP3, SAMD11, TGFB3, BMP5*), IGF (*IGF1, IGF2, IGF1R*), PDGF (*PDGFRA, PDGFRL, PDGFC*), and FGF (*FGF2, FGF7, FGFR1, FGFR4*) signaling pathways have been found to be highly expressed. We have previously reported that activation of TGF-β/Smad3 signaling is responsible for the bone marrow-derived macrophage to myofibroblast transition 21,52. It is highly possible that upregulation of TGF-β signaling may be involved in this BMPC-derived fibroblast to myofibroblast process. In addition, the expression of *KLF4* was found to be significantly upregulated during the early stage of BMPC-derived fibroblast to myofibroblast differentiation. This observation is consistent with previous findings in fibrotic lung in which an upregulation of *KLF4* is observed in PDGFR-β+ cells 44. Consistent with previous findings that the expression of *Meis1* increases specifically in kidney myofibroblasts during their differentiation and proliferation 60, we also detected that *Meis1* was upregulated during the process of BMPC-derived fibroblast to myofibroblast differentiation. Thus, it is likely that *MEIS1* may be involved in myofibroblasts differentiation and proliferation under pathology conditions. Furthermore, upregulation of Notch signaling (*HES1* and *NOTCH3*) was observed during the pericyte to myofibroblast differentiation. This is also consistent with a known role of Notch in renal fibrosis 61. Nevertheless, findings from the present study are largely observational and further mechanistic studies involved in this BMPC-derived myo-/fibroblast transition are needed.

In summary, circulating bone marrow-derived progenitor cells are a major source of ECM-producing cells with proinflammatory properties and may play a driving role in tissue fibrosis across a wide range of human fibrotic diseases. Targeting the molecular signaling mechanisms underlying the recruitment and differentiation of bone marrow-derived fibroblast precursors may serve as a novel therapeutic strategy for fibrosis. However, it should be pointed out that the lineage differentiation and regulatory mechanisms/pathways involving these BMPC-derived ECM-producing cells are largely unclear. In addition, fibrosis is the result of the interaction between a variety of cells. Thus, further research is needed to fully understand the cell-to-cell interactions and mechanisms underlying lineage differentiation with potential implications for targeted therapeutic interventions against fibrosis.

## Methods

### Data acquisition

Processed single-cell RNA sequencing of human failing heart from Rao* et al.* 33 is available from the Gene Expression Omnibus (GEO) with accession number: GSE145154. scRNA-seq data of 16 kidney allograft samples from Lamarthée *et al.* 27 is available from the European Nucleotide Archive (ENA) with accession number: PRJEB55286. Processed scRNA-Seq data of 5 kidney transplant biopsies from Malone* et al.* 28 is available from the GEO with accession number: GSE145927. Processed scRNA-seq data of 3 kidney biopsies from Suryawanshi *et al.* 29 is available from the GEO with accession number: GSE151671. Processed scRNA-seq data of fluorescence-activated cell sorting (FACS) sorted CD10^-^ human CKD samples from Kuppe *et al.* 13 is available from the Zenodo data archive with accession number: 4059315. Processed scRNA-seq data of human liver cirrhosis from Ramachandran *et al.* 30 is available from the GEO with accession number: GSE136103. Processed scRNA-seq data of idiopathic pulmonary fibrosis lung from Adams *et al.* 31 is available from the GEO with accession number: GSE136831. Processed scRNA-seq data of pulmonary fibrosis lung from Habermann *et al.* 32 is available from the GEO with accession number: GSE135893. Table S1 lists the studies used in this study.

### Single-cell RNA-seq data processing

The raw droplet-based 10x Genomics scRNA-seq data were processed using CellRanger (7.0.1 version, 10x Genomics) 62 to align with the human genome reference (GRCh38) and generate the gene × cell UMI matrix for each sample. The gene × cell UMI count matrices were used for cell clustering analysis with Seurat (version 4.1.0) 63. For the raw expression matrix, cells with less than 200 genes (UMI > 0) or over 25% UMI originating from the mitochondrial genome were identified as low-quality cells. After removing low-quality cells, the gene count matrix was normalized using log1p normalization. Next, the top 3000 highly variable genes were selected by the “FindVariableFeatures” function to perform principal component analysis. The Harmony 64 algorithm was utilized for the heart failure dataset to integrate samples from different patients by employing 30 principal components (PCs). Subsequently, 20 dimensions of PCs/harmony reduction were used to perform Louvain clustering and Uniform Manifold Approximation and Projection (UMAP)-based visualization with resolution = 0.6. Each cluster was annotated by an extensive literature review and searches for expression patterns of known markers. Analysis of differentially expressed genes (DEGs) among different cell types was performed with the FindAllMarkers function using log1p normalized data with adjusted *p* < 0.05 to confirm the identity of the annotated cell types. We also reviewed and cross-referenced the annotations and markers provided in the original publications to validate accurate annotations in each dataset.

### Stromal cells clustering

The stromal cells including fibroblast, myofibroblast, vSMC, pericyte and mesothelial were identified as ECM-producing cells by expression of ECM genes. These expression data of stromal cell subtypes across datasets were merged into a Seurat object 63. The gene count matrix was normalized using log1p normalization. Next, the top 3000 highly variable genes were selected by the “FindVariableFeatures” function to perform principal component analysis. The Harmony 64 algorithm was utilized to correct batch effects across different datasets by employing 30 PCs. Subsequently, 20 dimensions of harmony reduction were used to perform Louvain clustering and UMAP-based visualization with resolution = 0.6. Each cluster was annotated by an extensive literature review and searches for expression patterns of known markers. Analysis of DEGs among different cell types was performed with the FindAllMarkers function using log1p normalized data with adjusted *p* < 0.05.

### ECM-producing fibroblast and myofibroblast subclustering

The top 2000 highly variable genes for fibroblasts and myofibroblasts were selected by the “FindVariableFeatures” function in the Seurat 63 package to perform principal component analysis. The Harmony 64 algorithm was utilized to correct batch effects across different datasets by employing 30 PCs. Subsequently, 10 dimensions of harmony reduction were used to perform Louvain clustering a UMAP-based visualization with resolution = 0.6. Analysis of DEGs among different clusters was performed with the FindAllMarkers function using log1p normalized data with adjusted *p* < 0.05. Each cluster was annotated by discriminated markers of each cluster.

### Gene Ontology (GO) enrichment analysis

R package clusterProfiler 65 was used to characterize the biological functions of the combined gene list of differentially expressed genes of different cell subtypes. GO terms with a corrected *P* value of less than 0.05 were considered significantly enriched by differentially expressed genes. Dot plots were used to visualize enriched terms by the enrichplot R package.

### Gene score calculation

We scored the gene scores of each cell using 'AddModuleScore' from Seurat 63 with default parameters. ECM (collagens, glycoproteins, and proteoglycans) gene sets were obtained from the publication of Naba *et al.* 34. Hallmark gene sets (v7.4) were downloaded from MsigDB (https://www.gsea-msigdb.org/gsea/msigdb).

### Identification of recipient-derived or donor-derived cells

For kidney allograft samples from Suryawanshi *et al.* 29, fibroblasts from AK1 with the expression of X chromosome-specific gene *XIST* (female recipient) were identified as recipient-derived fibroblasts, while fibroblasts from AK2 with the expression of Y chromosome-specific gene *RPS4Y1* (male recipient) were identified as recipient-derived fibroblasts. For kidney allograft samples from Malone *et al.* 28, the sources of cells were acquired from public repository which were identified by single nucleotide variation (SNV).

### Discriminating the source of myo-/fibroblasts

The gene scores of circulating and resident fibroblasts for each cell were calculated using the feature genes of recipient-derived and donor-derived fibroblasts, respectively. This was achieved by employing the 'AddModuleScore' function from Seurat with default parameters. To determine the cutoff values for circulating and resident fibroblast gene scores, thresholds of 0 and 0.8 were applied based on their respective distributions.

### DEG analysis

The differential expression analysis between recipient-derived or donor-derived cells was carried out using the “FindMarkers” function, which is implemented in the Seurat package (version 4.1.0) 63 with default parameters.

### Cell trajectory inference

The Slingshot R package 66 was used for cell trajectory tree inference and pseudotime cell ordering inference based on the diffusion map projection. Myofibroblasts, fibroblasts and pericytes were used as input cells. Myofibroblast was set as an end cell cluster based on diffusion map projection and the reasonable expectation given our prior knowledge.

### Gene dynamics along pseudotime

The DEGs along pseudotime were detected by the “differentialGeneTest” in monocle (v2.24.1) R package 67 with the cutoff of *p* value < 1e-6. Gene clusters and expression heatmap were produced by ordering cells along the pseudotime using the “plot_pseudotime_heatmap” function. This function clusters genes using the k-means algorithm, and we set the number of gene clusters to three.

### Statistical analysis

The differences between the two groups were assessed using the Wilcoxon test, with *p*-value < 0.05 being considered statistically significant. Significance levels were indicated as * *p* < 0.05, ** *p* < 0.01, *** *p* < 0.001 and **** *p* < 0.0001. All data analysis and presentation were done using R (version 4.2.0).

## Supplementary Material

Supplementary figures.

Supplementary tables.
