# Supplementary material for: Single-Cell RNA-Sequencing Identifies Bone Marrow-Derived Progenitor Cells as a Main Source of Extracellular Matrix-Producing Cells Across Multiple Organ-Based Fibrotic Diseases
Source: Int J Biol Sci. 2024 Sep 16;20(13):5027–42. doi: 10.7150/ijbs.98839 (PMC11488580; doi:10.7150/ijbs.98839)
Supplement: Supplementary file 1 — Supplementary figures. [file ijbsv20p5027s1.pdf]

Figure S1. Features of ECM-producing stromal cells. The heatmap displays the expression of the top 20 marker genes of each cell type **(A)**, proteoglycan genes **(B)**, collagen genes **(C)** and glycoprotein genes **(D)** for each ECM-producing stromal cell type.

Figure S2. Features of myo-/fibroblasts subtypes. (A) UMAP shows the myo-/fibroblasts from different datasets. **(B)** The bar plot represents the cell proportion of the 6 myo-/fibroblasts subtypes among the 8 datasets. **(C)** The heatmap displays the expression of the top 10 marker genes of each myo-/fibroblasts subtype. **(D)** Violin plot displays the expression of selected feature genes in myo-/fibroblast subtypes.

Figure S3. Characteristics and marker gene expression in fibroblasts derived from peripheral circulating cells and from tissue-resident cells. (A) Bubble plots show expression of collagen, HSC, and MSC marker genes in myo-/fibroblast subtypes across datasets. Bubble size indicates the proportion of cells expressing each marker; color intensity represents the average expression level. **(B)** UMAP of cell clusters representing different cell types (left panel) and cells from three kidney biopsy tissues (right panel). PT, proximal tubular cells; IC, intercalated cells; LOH, loop of Henle; NK, natural killer cells; vSMC, vascular smooth muscle cells; HK-healthy kidney biopsy tissue; AK1 and AK2-allograft kidney biopsy tissues. **(C)** UMAP of cell clusters representing different cell types from human fetal bone marrow (left panel) and cells from different samples (right panel). MSC, mesenchymal stem cells; PreB, B cell progenitors; CLP, common lymphoid progenitors; pDC, plasmacytoid dendritic cells; NK, natural killer cells; PCW, post-conception weeks. (**D)** Bubble plot shows expression of canonical cell marker genes in each cell type in the human fetal bone marrow dataset. Bubble size indicates the proportion of cells of inferred cell type expressing each marker; color intensity represents the average expression level. **(E)** The circulating and resident gene scores of myo-/fibroblasts. **(F)** Boxplot shows the proportion of circulating and resident myo-/fibroblasts subtypes. Statistical analysis was performed using the Wilcoxon test. * $p < 0.05$, and ** $p < 0.01$.

Figure S1

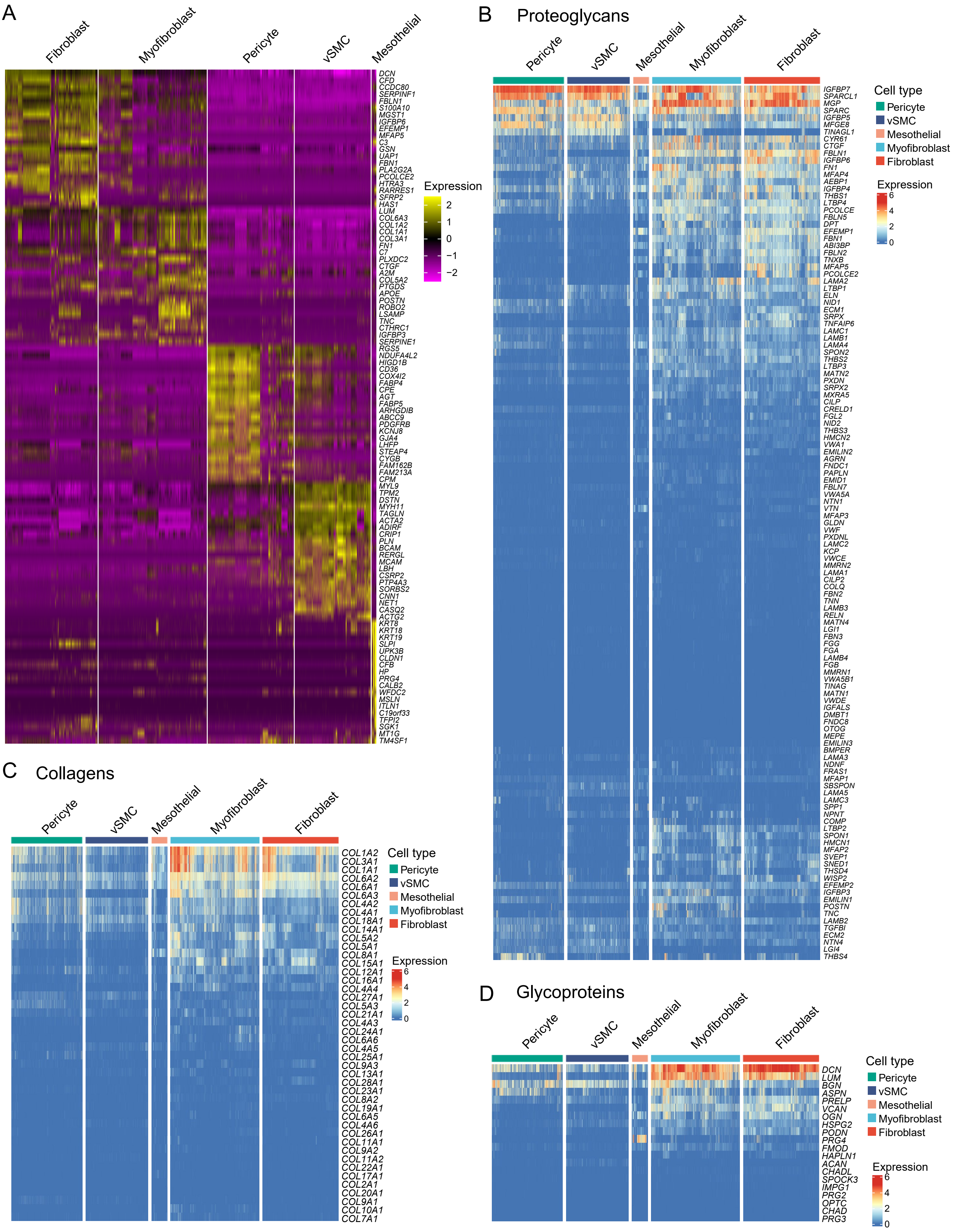
A
Fibroblast
Myofibroblast
Pericyte
vSMC
Mesothelial
DCN
CFD
CCDC80
SERPINF1
FBLN1
S100A10
MGST1
IGFBP6
EFEMP1
MFAP5
C3
GSN
UAP1
FBN1
PLA2G2A
PCOLCE2
HTRA3
RARRES1
SFRP2
HAS1
LUM
COL6A3
COL1A2
COL1A1
COL3A1
FN1
C7
PLXDC2
CTGF
A2M
COL5A2
PTGDS
APOE
POSTN
ROBO2
LSAMP
TNC
CTHRC1
IGFBP3
SERPINE1
RGS5
NDUFA4L2
HIGD1B
CD36
COX4I2
FABP4
CPE
AGT
FABP5
ARHGDIB
ABCC9
PDGFRB
KCNJ8
GJA4
LHFP
STEAP4
CYGB
FAM162B
FAM213A
CPM
MYL9
TPM2
DSTN
MYH11
TAGLN
ACTA2
ADIRF
CRIP1
PLN
BCAM
RERGL
MCAM
LBH
CSRP2
PTP4A3
SORBS2
CNN1
NET1
CASQ2
ACTG2
KRT8
KRT18
KRT19
SLPI
UPK3B
CLDN1
CFB
HP
PRG4
CALB2
WFDC2
MSLN
ITLN1
C19orf33
TFPI2
SGK1
MT1G
TM4SF1
Expression
2
1
0
−1
−2
B
Proteoglycans
Pericyte
vSMC
Mesothelial
Myofibroblast
Fibroblast
IGFBP7
SPARCL1
MGP
SPARC
IGFBP5
MFGE8
TINAGL1
CYR61
CTGF
FBLN1
IGFBP6
FN1
MFAP4
AEBP1
IGFBP4
THBS1
LTBP4
PCOLCE
FBLN5
DPT
EFEMP1
FBN1
ABI3BP
FBLN2
TNXB
MFAP5
PCOLCE2
LAMA2
LTBP1
ELN
NID1
ECM1
SRPX
TNFAIP6
LAMC1
LAMB1
LAMA4
SPON2
THBS2
LTBP3
MATN2
PXDN
SRPX2
MXRA5
CILP
CRELD1
FGL2
NID2
THBS3
HMCN2
VWA1
EMILIN2
AGRN
FNDC1
PAPLN
EMID1
FBLN7
VWA5A
NTN1
VTN
MFAP3
GLDN
VWF
PXDNL
LAMC2
KCP
VWCE
MMRN2
LAMA1
CILP2
COLQ
FBN2
TNN
LAMB3
RELN
MATN4
LGI1
FBN3
FGG
FGA
LAMB4
FGB
MMRN1
VWA5B1
TINAG
MATN1
VWDE
IGFALS
DMBT1
FNDC8
OTOG
MEPE
EMILIN3
BMPER
LAMA3
NDNF
FRAS1
MFAP1
SBSPON
LAMA5
LAMC3
SPP1
NPNT
COMP
LTBP2
SPON1
HMCN1
MFAP2
SVEP1
SNED1
THSD4
WISP2
EFEMP2
IGFBP3
EMILIN1
POSTN
TNC
LAMB2
TGFBI
ECM2
NTN4
LGI4
THBS4
Cell type
Pericyte
vSMC
Mesothelial
Myofibroblast
Fibroblast
Expression
6
4
2
0
C
Collagens
Pericyte
vSMC
Mesothelial
Myofibroblast
Fibroblast
COL1A2
COL3A1
COL1A1
COL6A2
COL6A1
COL6A3
COL4A2
COL4A1
COL18A1
COL14A1
COL5A2
COL5A1
COL8A1
COL15A1
COL12A1
COL16A1
COL4A4
COL27A1
COL5A3
COL21A1
COL4A3
COL24A1
COL6A6
COL4A5
COL25A1
COL9A3
COL13A1
COL28A1
COL23A1
COL8A2
COL19A1
COL6A5
COL4A6
COL26A1
COL11A1
COL9A2
COL11A2
COL22A1
COL17A1
COL2A1
COL20A1
COL9A1
COL10A1
COL7A1
Cell type
Pericyte
vSMC
Mesothelial
Myofibroblast
Fibroblast
Expression
6
4
2
0
D
Glycoproteins
Pericyte
vSMC
Mesothelial
Myofibroblast
Fibroblast
DCN
LUM
BGN
ASPN
PRELP
VCAN
OGN
HSPG2
PODN
PRG4
FMOD
HAPLN1
ACAN
CHADL
SPOCK3
IMPG1
PRG2
OPTC
CHAD
PRG3
Cell type
Pericyte
vSMC
Mesothelial
Myofibroblast
Fibroblast
Expression
6
4
2
0

Figure S2

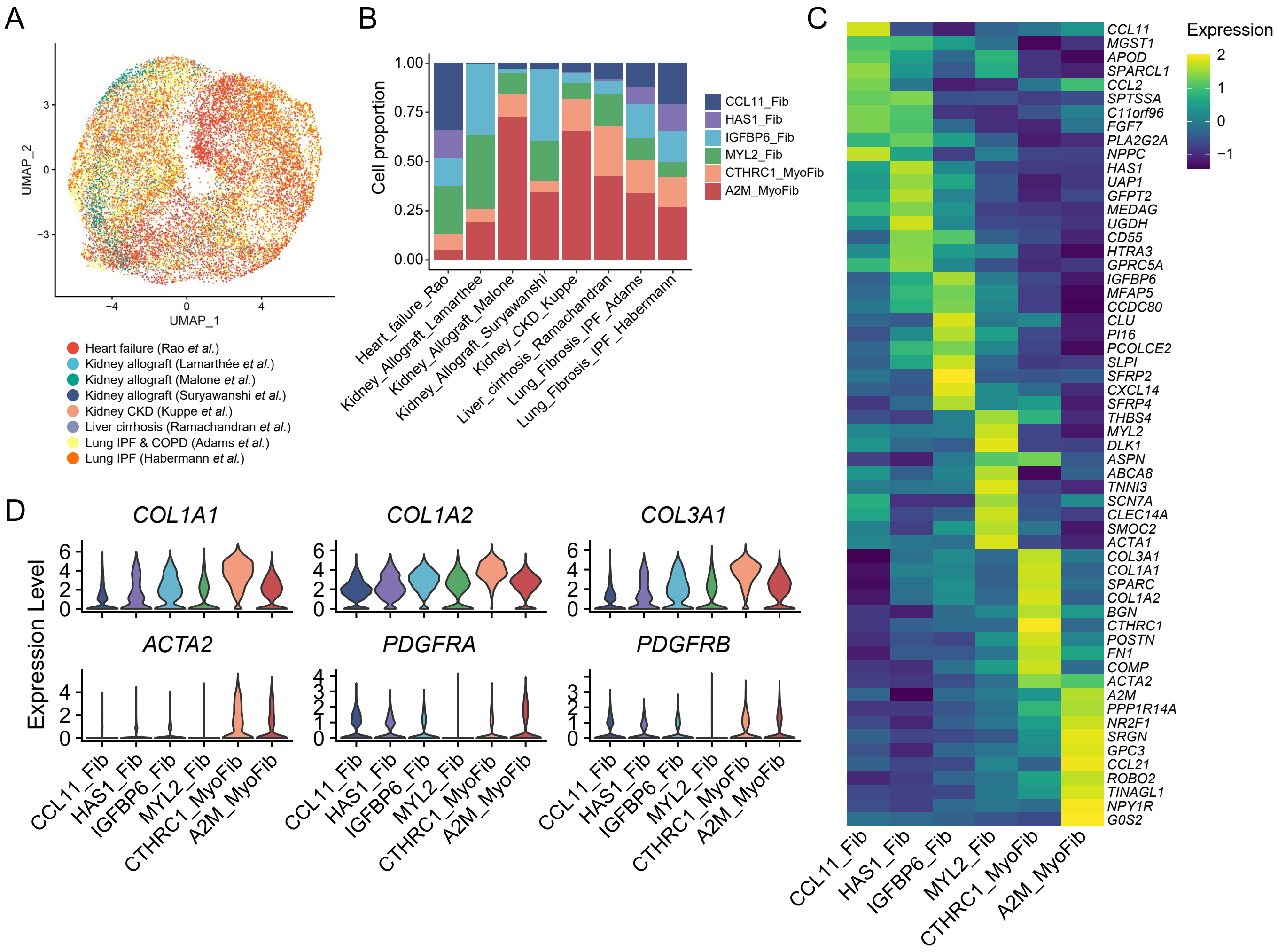
A
B
C
D
UMAP_2
UMAP_1
Heart failure (Rao et al.)
Kidney allograft (Lamarthée et al.)
Kidney allograft (Malone et al.)
Kidney allograft (Suryawanshi et al.)
Kidney CKD (Kuppe et al.)
Liver cirrhosis (Ramachandran et al.)
Lung IPF & COPD (Adams et al.)
Lung IPF (Habermann et al.)
Cell proportion
Heart_failure_Rao
Kidney_Allograft_Lamarthee
Kidney_Allograft_Malone
Kidney_Allograft_Suryawanshi
Kidney_CKD_Kuppe
Liver_cirrhosis_Ramachandran
Lung_Fibrosis_IPF_Adams
Lung_Fibrosis_IPF_Habermann
CCL11_Fib
HAS1_Fib
IGFBP6_Fib
MYL2_Fib
CTHRC1_MyoFib
A2M_MyoFib
Expression
CCL11
MGST1
APOD
SPARCL1
CCL2
SPTSSA
C11orf96
FGF7
PLA2G2A
NPPC
HAS1
UAP1
GFPT2
MEDAG
UGDH
CD55
HTRA3
GPRC5A
IGFBP6
MFAP5
CCDC80
CLU
PI16
PCOLCE2
SLPI
SFRP2
CXCL14
SFRP4
THBS4
MYL2
DLK1
ASPN
ABCA8
TNNI3
SCN7A
CLEC14A
SMOC2
ACTA1
COL3A1
COL1A1
SPARC
COL1A2
BGN
CTHRC1
POSTN
FN1
COMP
ACTA2
A2M
PPP1R14A
NR2F1
SRGN
GPC3
CCL21
ROBO2
TINAGL1
NPY1R
G0S2
Expression Level
COL1A1
COL1A2
COL3A1
ACTA2
PDGFRA
PDGFRB

Figure S3

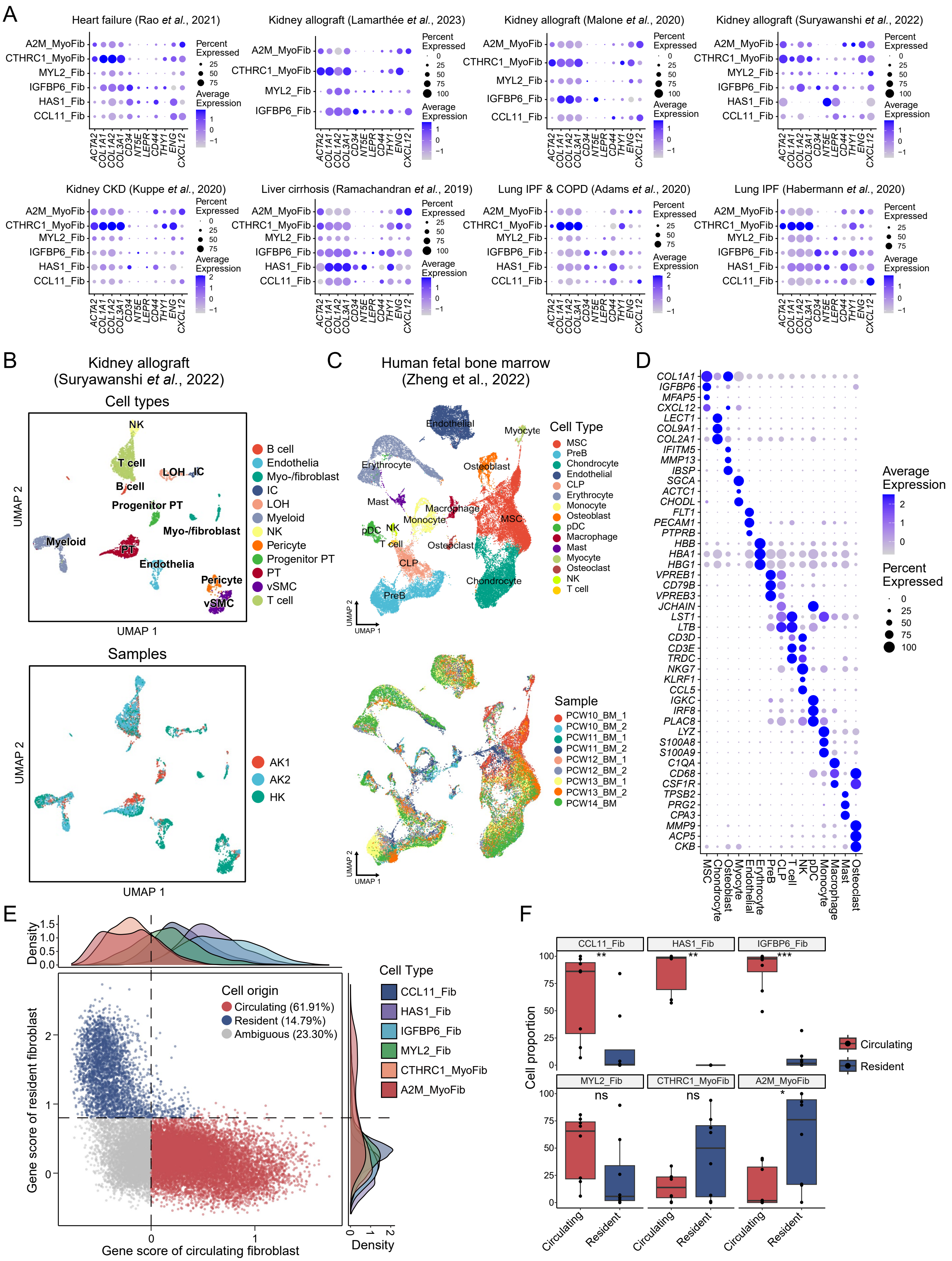

A
Heart failure (Rao et al., 2021)
Kidney allograft (Lamarthée et al., 2023)
Kidney allograft (Malone et al., 2020)
Kidney allograft (Suryawanshi et al., 2022)
Kidney CKD (Kuppe et al., 2020)
Liver cirrhosis (Ramachandran et al., 2019)
Lung IPF & COPD (Adams et al., 2020)
Lung IPF (Habermann et al., 2020)
A2M_MyoFib
CTHRC1_MyoFib
MYL2_Fib
IGFBP6_Fib
HAS1_Fib
CCL11_Fib
ACTA2 COL1A1 COL1A2 COL3A1 CD34 NT5E LEPR CD44 THY1 ENG CXCL12
Percent Expressed
Average Expression
B
Kidney allograft (Suryawanshi et al., 2022)
Cell types
Samples
UMAP 1
UMAP 2
B cell
Endothelia
Myo-/fibroblast
IC
LOH
Myeloid
NK
Pericyte
Progenitor PT
PT
vSMC
T cell
AK1
AK2
HK
C
Human fetal bone marrow (Zheng et al., 2022)
Cell Type
MSC
PreB
Chondrocyte
Endothelial
CLP
Erythrocyte
Monocyte
Osteoblast
pDC
Macrophage
Mast
Myocyte
Osteoclast
NK
T cell
Sample
PCW10_BM_1
PCW10_BM_2
PCW11_BM_1
PCW11_BM_2
PCW12_BM_1
PCW12_BM_2
PCW13_BM_1
PCW13_BM_2
PCW14_BM
D
Average Expression
Percent Expressed
COL1A1 IGFBP6 MFAP5 CXCL12 LECT1 COL9A1 COL2A1 IFITM5 MMP13 IBSP SGCA ACTC1 CHODL FLT1 PECAM1 PTPRB HBB HBA1 HBG1 VPREB1 CD79B VPREB3 JCHAIN LST1 LTB CD3D CD3E TRDC NKG7 KLRF1 CCL5 IGKC IRF8 PLAC8 LYZ S100A8 S100A9 C1QA CD68 CSF1R TPSB2 PRG2 CPA3 MMP9 ACP5 CKB
MSC Chondrocyte Osteoblast Myocyte Endothelial Erythrocyte PreB CLP T cell NK pDC Monocyte Macrophage Mast Osteoclast
E
Density
Cell origin
Circulating (61.91%)
Resident (14.79%)
Ambiguous (23.30%)
Gene score of resident fibroblast
Gene score of circulating fibroblast
Cell Type
CCL11_Fib
HAS1_Fib
IGFBP6_Fib
MYL2_Fib
CTHRC1_MyoFib
A2M_MyoFib
F
CCL11_Fib **
HAS1_Fib **
IGFBP6_Fib ***
MYL2_Fib ns
CTHRC1_MyoFib ns
A2M_MyoFib *
Cell proportion
Circulating
Resident
